# Mapping Lyme Disease Incidence for Diagnostic and Preventive Decisions, Maryland

**DOI:** 10.3201/eid0804.000413

**Published:** 2002-04

**Authors:** Christina Frank, Alan D. Fix, César A. Peña, G. Thomas Strickland

**Affiliations:** * University of Maryland Baltimore, Baltimore, MD, USA; † Maryland Department of Health and Mental Hygiene, Baltimore, MD, USA.

**Keywords:** Key words: Lyme disease, geographic distribution, epidemiology, diagnosis, prevention, vaccine

## Abstract

To support diagnostic and preventive decision making, we analyzed incidence of Lyme disease in Maryland on the zip code level. Areas of high incidence were identified on the Upper Eastern Shore of the Chesapeake Bay and in counties north and east of Baltimore City. These latter foci, especially, are not visible when mapping Lyme disease on the county level.”

 Lyme disease (LD) is a multisystem infectious and inflammatory disease resulting from infection with the spirochete *Borrelia burgdorferi*. It is by far the most commonly reported vector-borne disease in the United States and is transmitted by the bite of infected *Ixodes scapularis* ticks (*1*). In the United States, areas at high risk for the disease focally occur in temperate wooded habitats sustaining *B. burgdorferi*’s small mammalian hosts, predominantly the white-footed mouse (*Peromyscus leucopus),* as well as the preferred mating place for adult ticks,on the white-tailed deer (*Odocoilus virginianus*) (*2*).

 We have reported the incidence of LD for Maryland by county (*3*). Glass and colleagues developed a detailed LD risk map of Baltimore County by using environmental risk factors within a geographic information system (GIS) (*4*). The objective of our report is to show areas of high incidence of LD on a level more detailed than the standard reporting by county. The next most detailed geographic boundary system for which population data are available is the zip code level. It provides intermediate detail between counties and census blocks. The zip code also allows incidence calculations based on census population figures and, by relying on postal address, uses a feature of geographic reference very commonly available to state health departments n Maryland and elsewhere. We believe this level of detail in mapping the focal distribution of LD will improve decision-making regarding diagnoses, personal and community interventions, and cost-effective use of vaccine.

## The Study

Included in our report were all cases meeting the national surveillance case definition for LD (*5*) reported to the Maryland Department of Health and Mental Hygiene (DHMH) with a known date of onset from 1993 through 1998 and a residential zip code mailing address.

All cases were referenced to zip code of residence. Demographic data from the 1990 census are publicly available for the zip code level (*6*). In 1990, population figures for Maryland zip codes ranged from 39 to 56,594 (median 3,042). To obtain larger units of population for more stable estimates of incidence, small zip codes were combined with the next smallest neighboring zip codes until the aggregated zip code area (AZCA) reached the size of >600 residents or more. Annual average incidence per zip code or AZCA was calculated as the average number of cases from 1993 through 1998 per 100,000 population. One hundred six small-population zip codes were combined with others to give 50 AZCAs, ranging in size from 616 to 51,683 (median 1,791). Most AZCAs were located in western Maryland (non disease-endemic area) and the Eastern Shore (highly endemic area). The 1990 census did not contain population data for 15 zip codes, so their incidence could not be calculated.

Analysis and data management were performed with Epi-Info version 6.04 and Microsoft EXCEL version 7.0; maps were created with ArcInfo (ESRI, Redlands, CA).

A total of 2,399 cases reported to the DHMH with a known date of onset from 1993 through 1998 met the national surveillance case definition for LD. This report includes the 2,371 (99%) patients for whom mailing addresses were available. Cases were reported from 344 (80%) of 431 zip codes. Only 6 of the 33 zip codes from western Maryland (Garrett, Allegheny, Washington, and Frederick Counties) reported cases of LD during the study period.

Two areas of high incidence are evident: the Upper Eastern Shore (Cecil, Kent, Queen Anne’s, Caroline, and Talbot Counties), and focal areas north and east of Baltimore City in Baltimore and Harford Counties (Figure). This latter area is part of an arc of increased incidence, from Montgomery County in the south, extending northeast through Howard, southeastern Carroll, Baltimore, and Harford Counties into Cecil County. This arc parallels the “fall line,” the topographic boundary where the Coastal Plains meet the Piedmont and land elevations begin to rise towards the Appalachian Mountains.

**Figure:**
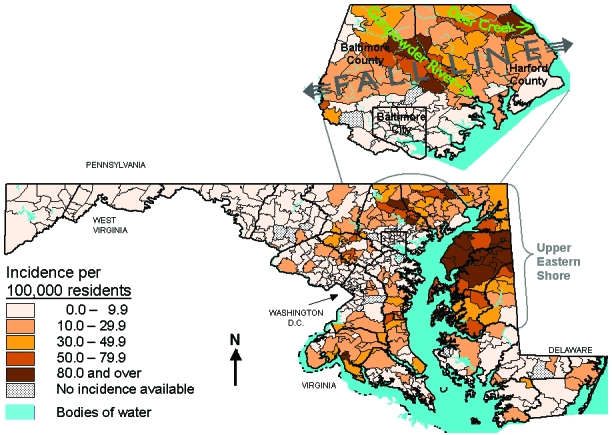
Average annual incidence of Lyme disease by zip code, Maryland, 1993–1998.

In Baltimore County, the area with the highest incidence extends along the vegetational corridors bordering the Gunpowder Falls river system and associated reservoirs. In Harford County, a similar but less confined linear pattern follows the runs of Broad Creek and Deer Creek. On the Upper Eastern Shore high incidence is more uniformly observed, without any clear topographic pattern. The scattered zip codes on the Lower Eastern and Lower Western Shores that show higher incidence than surrounding areas are all AZCAs with rather small populations and resultant wide confidence intervals for their incidence estimates.

## Conclusions

The detailed mapping of LD in Maryland identifies an area of high LD incidence north of Baltimore City that is not be apparent when mapping on the county level (*3*). When analyzed by counties, focal high incidence along Gunpowder River and Deer Creek is diluted by adjacent areas of lower incidence, especially the northern inner suburbs of Baltimore City with their comparatively urban environment. These foci are aligned along the larger rivers and creeks in an environment that is ideal for transmission of the disease. Within the floodplain and on valley slopes of the rivers descending from the Piedmont lie corridors of forest and brush, cutting through rural and suburban areas. Farms, estates, individual houses, and housing developments lie within and adjoin these ideal tick habitats. Local outdoor recreation is widspread. The extent of the high-risk area in Baltimore County is congruent with Glass’s detailed GIS results (*4*).

In contrast, Maryland’s Upper Eastern Shore, a rural area situated entirely in the Coastal Plain with an ideal tick habitat, has uniformly high LD incidence. However, southern Maryland (north of the Potomac River) and the Lower Eastern Shore have a low incidence of LD. This correlates with limited ecologic data showing much lower *B. burgdorferi* infection rates in *I. scapularis* in southern Maryland and the Lower Eastern Shore than in the Upper Eastern Shore (*7*,*8*). Almost no LD was reported in western Maryland from Frederick County westward; this virtual absence is consistent with the low prevalence of *I. scapularis* and low *B. burgdorferi* infection rates in this tick species, despite an abundance of rodents and deer in this mountainous region (*7*,*8*).

Spot-mapping of LD cases is useful for tracking LD transmission but can be misleading about incidence because population density is not taken into consideration. On a spot map, based on the absolute number of cases, a sparsely populated area with high LD incidence may be indistinguishable from another area with high population density and low incidence. Incidence figures for counties containing both highly urban and highly rural areas are likely not representative of the rural areas because of the concentration of population in the urban part. Health-care providers must appreciate the fact that, for instance, more cases of LD are reported from Baltimore County (population ~1 million) than from Cecil County (population 20,000), even though the county-level incidence of LD is much higher in Cecil County.

Characterization of LD incidence on the zip code level is feasible using data collected routinely by local health departments. Zip code level data provide more detailed information than county level data and require less data and effort than GIS risk modeling based on vegetation parameters and tick distribution (*4*,*9*–*11*). Although LD risk-mapping based on prevalence of infection in ticks would be the most accurate method (*12*), tick data are often unavailable, out-of-date, costly, and difficult to collect. A potential limitation of our report is that incidence has been referenced to residential addresses, whereas patients may have been infected elsewhere. However, residence in an LD-endemic area is a well-recognized risk factor for infection (*12*). Many studies have reported that patients with LD usually believe they were infected at their homes, places of work, or some nearby recreational site (*13*–*17*). A calculated entomologic risk index showed a strong positive relation with the geographic LD case rate in Rhode Island (*18*). Most (58%) of our study participants who remembered a tick bite believed it occurred at or near their place of residence; an additional 21% were bitten during recreation and 9% at work. Patients in high incidence areas were more likely to report a tick bite near their home than were those living in more urban areas or in western Maryland. Referencing LD cases to their residence is a useful proxy for the actual place patients acquired a tick bite.

Knowledge of focal LD risk distilled from mapping on the zip code level is of value to the general public. It can focus efforts to reduce tick exposure and increase motivation to use appropriate preventive measures when tick exposure is unavoidable. Such mapping can also aid health-care providers in assessing the likelihood of a particular patient’s having LD (*19*).

Meltzer et al. estimated how much the cost-effectiveness of LD vaccination depends on individual risk (*20*). Mapping LD incidence in detail complements the Centers for Disease Control and Prevention’s (CDC’s) recommendation that the LD vaccine be administered based on residential, occupational, and recreational risk assessment (*21*). The CDC report recognizes the need to “develop maps of geographic distribution of LD with improved accuracy and predictive power” beyond the county-based national LD risk map. This level of detail would aid the “Healthy People 2010” goal of LD prevention through targeted vaccination (*22*).
